# Relationships between climate and growth of *Gymnocypris selincuoensis* in the Tibetan Plateau

**DOI:** 10.1002/ece3.1463

**Published:** 2015-03-24

**Authors:** Juan Tao, Yifeng Chen, Dekui He, Chengzhi Ding

**Affiliations:** 1 Laboratory of Biological Invasion and Adaptive Evolution, Institute of Hydrobiology, Chinese Academy of SciencesWuhan, 430072, China; 2 University of Chinese Academy of SciencesBeijing, 100049, China; 3 Australian Rivers Institute, Griffith UniversityNathan, Queensland, 4111, Australia; 4 Asian International Rivers Center, Yunnan UniversityKunming, 650091, China; 5 College Animal Science &Technology, Yunnan Agriculture UniversityKunming, 650201, China

**Keywords:** Climate change, dendrochronology, fish growth, *Gymnocypris selincuoensis*, Tibetan plateau

## Abstract

The consequences of climate change are becoming increasingly evident in the Tibetan Plateau, represented by glaciers retreating and lakes expanding, but the biological response to climate change by plateau–lake ecosystems is poorly known. In this study, we applied dendrochronology methods to develop a growth index chronology with otolith increment widths of Selincuo naked carp (*Gymnocypris selincuoensis*), which is an endemic species in Lake Selincuo (4530 m), and investigated the relationships between fish growth and climate variables (regional and global) in the last three decades. A correlation analysis and principle component regression analysis between regional climate factors and the growth index chronology indicated that the growth of *G. selincuoensis* was significantly and positively correlated with length of the growing season and temperature-related variables, particularly during the growing season. Most of global climate variables, which are relevant to the Asian monsoon and the midlatitude westerlies, such as El Nino Southern Oscillation Index, the Arctic Oscillation, North Atlantic Oscillation, and North America Pattern, showed negative but not significant correlations with the annual growth of Selincuo naked carp. This may have resulted from the high elevation of the Tibetan Plateau and the high mountains surrounding this area. In comparison, the Pacific Decade Oscillation (PDO) negatively affected the growth of *G. selincuoensis*. The reason maybe that enhancement of the PDO can lead to cold conditions in this area. Taken together, the results indicate that the Tibetan Plateau fish has been affected by global climate change, particularly during the growing season, and global climate change likely has important effects on productivity of aquatic ecosystems in this area.

## Introduction

Climate change has become an important scientific and environmental issue worldwide. Climate change substantially affects a broad range of organisms at different levels of organization and threatens biodiversity of various geographical distributions (Carpenter et al. 1992; Walther et al. 2002; Watson 2003; Dawson et al. 2011). The Tibetan Plateau is particularly sensitive to the effects of climate change and serves as a sentinel site to monitor global climate change (Liu and Chen 2000; Du et al. 2004; Xu et al. 2009). This area is rich in water resources that supply water to >20% of the global population (Haeberli et al. 1989; Immerzeel et al. 2010; Ma et al. 2011). Glacial retreat, permafrost degradation, and changes in the precipitation regime are increasingly obvious in this area and primarily affect the hydrological and physicochemical characteristics of plateau waters (Wang et al. 2007; Yao et al. 2012). Lake expansion is pervasive here. A total of 73 lakes have expanded to >1.0 km^2^, and 30 lakes are newly formed in the Tibetan Plateau during the past several decades (Ma et al. 2011). Lake Selincuo (88°50″–89°40″E, 31°50″–32°10″N; 4530 m), which is the largest lake in Tibet, increased 39.39% by area, from 1666.96 km^2^ in 1976 to 2323.6 km^2^ in 2009 (Meng et al. 2012). However, it is unknown how these aquatic ecosystems respond to climate change (Wischnewski et al. 2011).

Defining climate–growth relationships offers predictive ability to help forecast the consequences of future climate change (Black et al. 2008). However, indirect records of aquatic system growth, in which time series are of comparable length, are fairly uncommon and arduous or expensive to obtain (Gillanders et al. 2012). By applying dendrochronology (tree-ring) technology to develop multidecade, precisely dated chronologies of growth from calcified tissues such as bivalve shells and fish otoliths, scientists can address the relationships between aquatic taxa growth and climate change (Strom et al. 2004; Black et al. 2005; Rypel 2009; Matta et al. 2010). In return, this method can also be utilized to reconstruct environmental factors (Schöne et al. 2004; Strom et al. 2004; Black et al. 2009). They reveal these long-term relationships between fish growth are essential in understanding the change of natural mortality, biomass, and catch (Rountrey et al. 2014; ). Despite increasing interest in developing chronologies for aquatic organisms, this potential data source remains greatly underutilized (Black 2009; Morrongiello et al. 2012). A diversity of species, ecosystems, and locations for which chronologies exist can be utilized for this purpose (Black et al. 2008).

In this study, we choose Lake Selincuo as our study area, not only because it has expanded dramatically but also because it is an endorheic lake, which can reveal climate change (McCarthy 2001), and has been rarely interfered with man-made activities. The Selincuo naked carp *Gymnocypris selincuoensis* (Chen et al.) was also carefully selected as a study species. One reason is that this species is the only multidecadal longevity fish with limited distribution in Lake Selincuo. Its lifespan often exceeds 30 years old; thus, it is considered an ideal candidate for chronology and climate–growth relationships analyses (Chen et al. 2002b; Rypel 2009). This choice is also related to the clarity of annuli on otoliths and the periodicity of annual increments on otoliths as demonstrated by Chen et al. (2002b). A third factor for choosing Selincuo naked carp was based on a long-term consideration. As other fish in the subfamily Schizothoracinae are the predominant vertebrate group in the aquatic systems of the Tibetan Plateau (Cao et al. 1981; Yue 1998), successful application of dendrochronology to Selincuo naked carp could be a guide for more extensive studies in the future.

We utilized Selincuo naked carp to explore the climate change effects on fish in the Selincuo area using a dendrochronological approach in this study. Our objectives were to (1) evaluate the suitability of *G. selincuoensis* otoliths for chronology development and (2) establish growth–climate relationships by correlating the chronology with local and global climate factors. Such data will help characterize the climate factors to which *G. selincuoensis* is most vulnerable and will assist in disentangling the environmental variables associated with fish growth. Due to the high-clarity annuli on *G. selincuoensis* otolith sections and large temperature variation in a typical year in this area, our first prediction is that the otolith of *G. selincuoensis* is suitable to constructing chronology. We also predict that local temperature and other thermal-related factors significantly affected their growth, especially during the growing seasons. For the global-scale exploration, our hypothesis is that the effect of global climate variables on fish growth is not obvious and significant, mainly because of the obstructing of high mountains around the study area.

## Materials and Methods

A total of 544 specimens used in this study were caught from Lake Selincuo by triple gillnets and cast nets in 1997, 2010, and 2011 (Table1). All fish were collected after April of the collection year to ensure that most of the latest annuli were completed (Chen et al. 2002b). Conventional biological measurements were taken on fresh specimens in 2010 and 2011, and the range in total specimen length was 42–464 mm (Table1). For specimens from year 1997, we lost the range information of individual size. Otoliths were removed from the utricle, cleaned with water, dried, and stored in a labeled polythene tube. The otoliths were brought back to the laboratory to prepare transverse sections as described in detail in Li et al. (2009).

**Table 1:** Details of *Gymnocypris selincuoensis* samples used in this study

Date	#Fish	Total length (mm)	Age range (Years)
July, 1997	54	/	6–34
May and June, 2010	331	42–464	2–30
November, 2010	87	132–460	5–38
May, 2011	72	183–398	8–22

Then the “list year” technique was used to visually cross-date the sections (Yamaguchi 1991). Age was subtracted from the year of collection to determine the spawn year, so that every growth increment could be accurately assigned the correct calendar year. Each increment is comprised of an opaque summer growth zone and a translucent winter growth zone (Chen et al. 2002c). Only sections with clear annuli were utilized for further analysis. Those too opaque or translucent to read were excluded. Every section's age was read independently by two skilled viewers using an optical microscope. If the two viewers diverged in their interpretation of a section and could not reach agreement in a subsequent re-examination of the section, the section was eliminated from further analysis.

**Figure 1:**
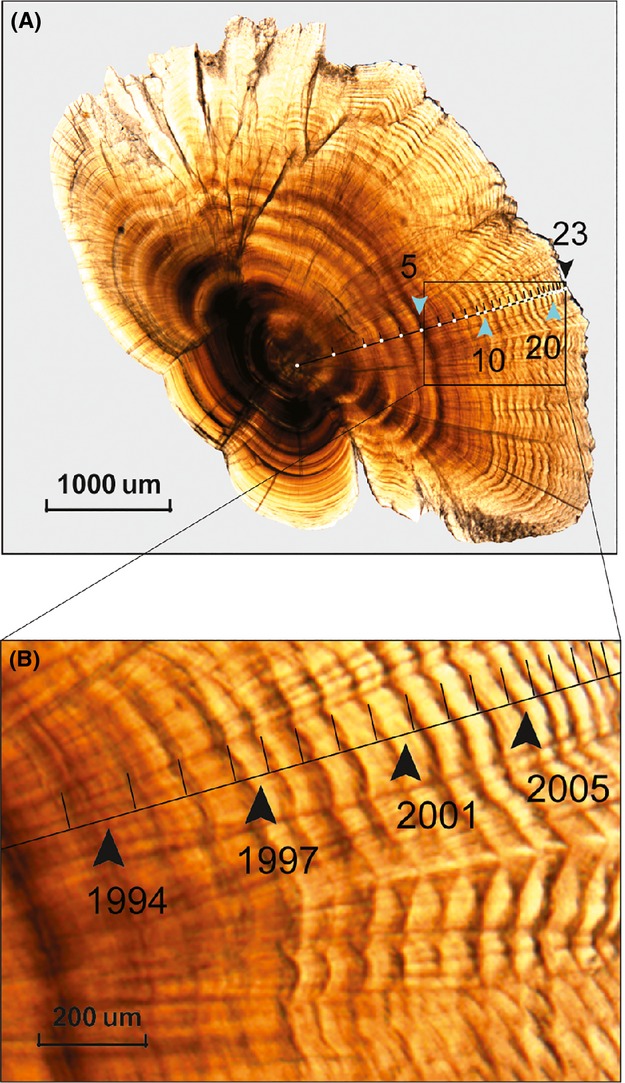
(A) Sectioned otolith of *Gymnocypris selincuoensis*, showing the annuli and axis (the line) of measurement; (B) Detail on enlarged scale of the oblong area on (A).

Otolith sections were photographed with a digital camera (MicroPublisher 5.0 Real Time Viewing) attached to a microscope under 40–100× magnification (Olympus BX51; Tokyo, Japan) according to their size. The annual growth increments were measured using an image analyzing software (Zhu et al. 2002). We commenced measurements at the most recent completed growth increment for each specimen and worked backwards according to calendar year. Measurements were taken along the primary growth axis to minimize variation (Chen et al. 2002a); in most cases, this was a straight line from the dorsal to the focus (Fig.1). Because the reproductive period of Selincuo naked carp is April–August (He et al. 2001), and the distance between the primordium and the first annulus depends on the hatch date of the individual, increment size for the first year of life (age 0+ years) was not measured.

After the measurements were completed, visual cross-dating was statistically checked using COFECHA software (developed by Richard L. Holmes and obtained from http://web.utk.edu/∼grissino/software). Only time series with at least six increments was retained for statistical cross-dating to ensure that the measured time series would be long enough for detrending. In COFECHA, each time series was fit with a cubic spline and a 50% frequency cutoff set at 14 years according to the mean series length and value of series intercorrelation. Each time series was standardized and then correlated with the average of all other standardized time series, and the average of all values was reported as the interseries correlation. Time series with nonsignificant values (*P* > 0.01) or lagged ±10 years in time and yielded a higher correlation coefficient was visually re-examined for errors. However, the ultimate decision about cross-dating should not be based on statistics alone. Instead, it should be made by visually examining each sample (Helama et al. 2006; Matta et al. 2010). Therefore, no cross-dating errors were found after re-examining the samples. Negative and poorly correlated (<0.1) samples with the average of all other standardized time series were excluded from analyses. The reason for omitting these samples is mainly because the main aim of this study is to explore the response of the main fish population in this lake. Insensitive individuals, individuals occupied occasional habitats, could mask important findings. Generally, COFECHA was used to assist to find cross-dating errors and negative or poorly correlated series in this study.

Once cross-dating verification was completed; the master growth index chronology was developed. The first step to establish a chronology is to detrend each original measurement time series with a negative exponential function. All detrended time series were then averaged into the master growth index chronology using the biweight robust mean to reduce the effects of outliers (Cook 1985). The master growth index chronology was developed using ARSTAN software (developed by Ed Cook and Paul Krusic, and obtained from http://web.utk.edu/∼grissino/software).

Pearson's correlation coefficients were used to detect the relationships between environmental variables and interannual growth as measured by the master growth index chronology. The environmental variables include lake area, local climate variables, and global climate variables. Local historical climate data were obtained from the hithermost meteorological station of the Tibet Meteorological Administration. The meteorological station is about 90 kilometers away in southeast of the sample site. Data included monthly mean air temperature, mean maximum air temperature, mean minimum air temperature, and total monthly precipitation. Historic water temperatures, which were considered essential to fish, were not available, so regional air temperature was used as a substitute. The growing season here is from April to October (Chen et al. 2002b; Yu et al. 2010). Data of annual snow accumulation days (ASAD) and lake area were provided by (Bian et al. 2010). Considering the nearly worldwide impacts of naturally occurring interannual climate oscillations such as the El Nino Southern Oscillation (ENSO)(Welsh et al. 2011), five global climate variables were chosen, including multivariate ENSO, the Arctic Oscillation (AO), the North Atlantic Oscillation (NAO), the North America Pattern (PNA), and the Pacific Decade Oscillation (PDO) as obtained from the National Climatic Data Center (http://www.ncdc.noaa.gov/oa/ncdc.html) and College of the Environment, University of Washington (http://jisao.washington.edu/pdo/). Correlation analyses were performed for all annually variables over both the current calendar year and the previous calendar year to discover any lagged relationships.

## Results

**Figure 2:**
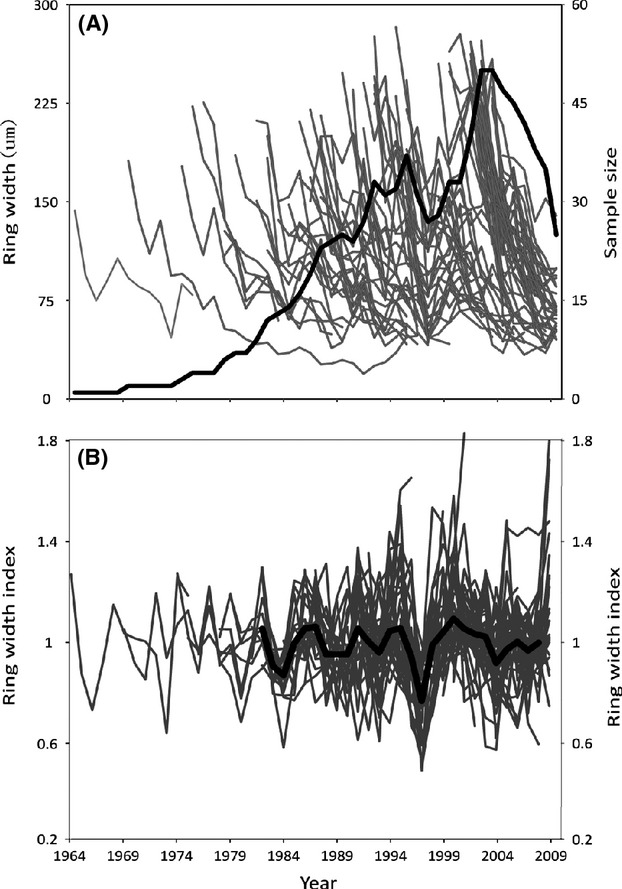
(A) Growth increment widths for 83 *Gymnocypris selincuoensis* are in light gray, the sample depth of each year is in bold; (B) *Gymnocypris selincuoensis* growth increment widths after detrending with a negative exponential function are in light gray; the biweight average of detrended measurement time series (the master growth index chronology) is in bold.

To fulfill the requirements of clarity and series length which were described in methods, 111 samples were left. Among them, only two errors were found by COFECHA and were corrected, and then, the other no-error series with a correlation index <0.1 (28 samples) were eliminated. This meant that 83 series were used to construct the master growth index chronology. The interseries correlation was 0.491, mean sensitivity was 0.192, mean series length was 11.0, and the autocorrelation was −0.004 (Table2). Thus, growth chronologies of individuals correlated well with growth chronologies of other individuals, and auto-regressive modeling was unnecessary. The final precisely dated *G. selincuoensis* chronology continuously spanned 1964–2009. The part of the chronology with a small sample size may have deviated strongly from the actual situation and could not be used to assess the relationship between *G. selincuoensis* growth and climate. The minimum sample depth of chronology is 10, covered from 1982 to 2009 (Fig.2A). Due to the absence of regional climate variables in 2009, the ensuing correlations analysis was conducted from 1982 to 2008 (Fig.2B).

**Table 2:** Properties of master growth chronology for *Gymnocypris selincuoensis* during 1964 and 2009

Sample size	Mean sensitivity	Interseries correlation	Mean series length (years)	Autocorrelation
83	0.192	0.491	11.0	−0.004

Sample Size: the number of time series used in the chronology development. Mean sensitivity: an index of high-frequency variability. Interseries correlation: an index of the synchrony of the time series used in the chronology development, which calculated by averaging correlation between each detrended time series and the average of all other detrended time series. Mean series length: average length of time series used in the chronology development. Autocorrelation: an index of the previous year effects on growth of current year.

**Figure 3:**
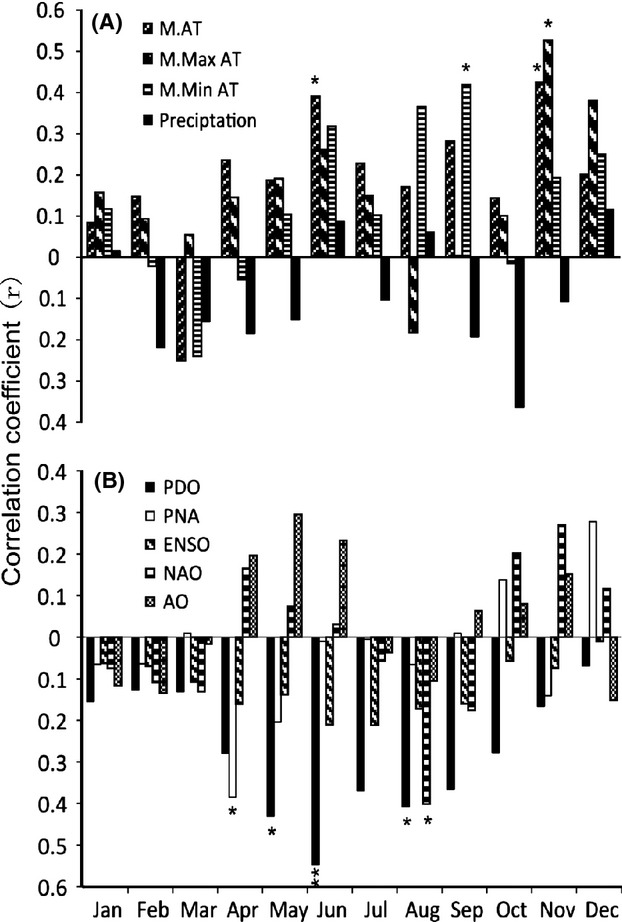
Correlations between master chronology and (A) monthly resolved local climate variables including mean air temperature (M. AT), mean maximum air temperature (M. Max AT), mean minimum air temperature (M. Min AT), and precipitation; (B) monthly resolved global climate variables including the El Nino-Southern Oscillation (ENSO), the Arctic Oscillation (AO), the North Atlantic Oscillation (NAO), the North America Pattern (PNA), and the Pacific Decade Oscillation (PDO).

Based on Pearson's correlation analysis, no effects of lake expansion can be reflected *G. selincuoensis* ring width index changes (*r* = 0.059, *P* = 0.769). Locally, the annual growth of *G. selincuoensis* was significantly affected by temperature- and thermal-related variables but with no lagged influence. Generally, although not always significantly correlated, growth width index of *G. selincuoensis* was increasing with temperature and decreasing with precipitation (Fig.3A). More specifically, the *G. selincuoensis* chronology was significantly and positively correlated with mean annual maximum air temperature (*r* = 0.415, *P* = 0.031) and mean growing season air temperature (*r* = 0.430, *P* = 0.037). In comparison, the ASAD had an obvious negative influence (*r* = −0.496, *P* = 0.009) on *G. selincuoensis* ring width index. A detailed analysis on the monthly effects of these local climate variables (Fig.3A) found that the growth index of *G. selincuoensis* was more sensitive to local climate variables in growing season than nongrowing season. For examples, the *r* values for correlations were generally higher in growing season when compared to nongrowing season (Fig.3A), and all the significant correlations occurred in growing season or just after (in November).

**Figure 4:**
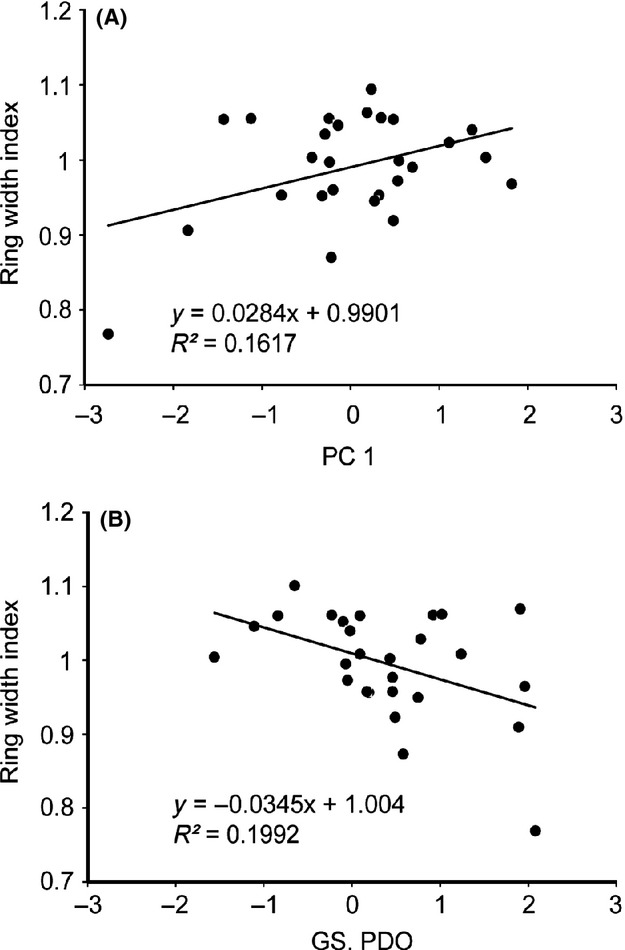
(A) Relationship between the *Gymnocypris selincuoensis* otolith growth increment chronology and the leading principal component (PC1) of the nine local climate variables; (B) Linear regression of growing season Pacific Decade Oscillation (GS. PDO) on the master chronology of *G. selincuoensis*.

The ASAD was significantly (*P* < 0.05) correlated with all air temperature variables (mean, maximum, and minimum) annually and in growing season, as well as some between temperature factors and precipitation factors (data not shown). To avoid problems with collinearity and to best capture overall local climate variables with respect to them, principal components regression (PCR) was used to better relate local climate to the *G. selincuoensis* chronology. The ASAD, temperature, and precipitation factors annually and of growing season, that is, nine climate variables in total, were first entered into principal components analysis, and the leading component (PC1) captured 56.2% of the variability in these nine climate variables (eigenvalue = 5.1). The second component captured 25.5% of the variability in these climate data (eigenvalue = 2.3). All the rest components were dropped from further analysis based on an eigenvalue threshold of 1. A multiple stepwise regression (*P* < 0.05 to enter) was performed for *G. selincuoensis*, and only the temperature principal component was significantly (*P* = 0.038) correlated to the chronology and explained 16.17% variance (Fig.4A). Thus, the positive relationships between principal components and the chronology indicated that warm air temperature and less ASAD were favorable for growth of *G. selincuoensis*.

On a global vision, only growing season PDO was found significantly (*P* = 0.019) and negatively (−0.447) affected the annual growth of *G*. *selincuoensis* among the global climate factors in annual or growing season scale. No lagged effect was found. Similar to the local scale analysis above, monthly resolved analysis reflected that the *G. selincuoensis* growth was strongly related to these global variables during growing season when compared to nongrowing season (Fig.3B). In contrast to the results of local scale analysis, most of the monthly global climate variables were negatively correlated to *G. selincuoensis* chronology (Fig.3B). Five of their correlations to *G. selincuoensis* were significant, namely April PNA (*r* = −0.385, *P* = 0.047), May PDO (*r* = −0.43, *P* = 0.035), June PDO (*r* = −0.547, *P* = 0.003), August PDO (*r* = −0.407, *P* = 0.047), and August NAO (*r* = −0.401, *P* = 0.038). A simple linear regression showed that growing season PDO can explain 19.92% variance of the ring width index of *G. selincuoensis*, which is a litter bit higher than the interpretability of PC1 in local PCR analysis.

## Discussion

### Suitability of *G. selincuoensis* otoliths for development of a chronology

The chronology developed in the present study is the highest inhabited yet developed for any fish species and could provide a basic understanding of the relationship between climate change and growth of fish in plateau regions. We effectively overcame the drawback of short longevity in freshwater fish and successfully constructed a nearly 30-year chronology with a minimum sample depth over 10 through multiple sampling. Eighty-three series were involved in constructing the chronology, and the sample size was larger than that in similar studies (Black et al. 2005, 2008; Rypel 2009). The comparable value of mean sensitivity (0.192) with other studies also revealed that Selincuo naked carp are sensitive to environmental change. Due to the relatively large sample size and long mean series length (11.0), the interseries correlation, which is an index of synchrony among all series, was slightly lower than that in similar studies (Black et al. 2005, 2008; Rypel 2009). Nevertheless, the interseries correlation and mean sensitivity sufficiently indicate that the *G. selincuoensis* otolith was suitable for the development of a chronology.

### Relationships between local climate factors and growth of *G. selincuoensis*

As our predictions, one interesting finding of this study is that the growth of *G. selincuoensis* was increasing along with the warming of environment conditions. This can be reflected and supported by the results related to temperature and precipitation (Fig.3A). Precipitation increase means substantial decrease in temperature in this area. Temperature is the principal contributing factor to annual growth of Selincuo naked carp as showed in PCR analysis (Fig.4A). Increasing temperature could accelerate the growth of Selincuo naked carp. The daily otolith increment width is proportional to somatic growth (Weisberg 1993; Oozeki and Watanabe 2000). Both the daily width increment and somatic growth rate of Selincuo naked carp larva increase with temperature during growing season (Ding 2012). Increment width becomes 2.26 times wider when water temperature increases by 10°C (Ding 2012). Numerous studies have shown that fish growth accelerates as temperature increases (Guyette and Rabeni 1995; Rypel 2009; Morrongiello et al. 2011). Lower temperature reduces fish feeding frequency and digestion rate (Brett and Higgs 1970; Salvatore et al. 1987; Marchand et al. 2002). Furthermore, temperature could have indirect influences by affecting the productivity of other species in the ecosystem (Gillanders et al. 2012).

Another finding in this study is, unlike other studies, the annual growth of Selincuo naked carp was particularly sensitive to climate variables and length of growing season rather than nongrowing season. Firstly, high *r* values of correlations between these variables and the chronology were mainly found in growing season (Fig.3A). Secondly, the growth index chronology of *G. selincuoensis* was significantly and negatively correlated with ASAD in the area. Due to the high elevation of Selincuo, this area often has a relatively long winter with low air temperature and a large area of snow cover. The length of the frozen lake period in the Tibetan Plateau is proportional to local ASAD. In other words, more ASAD means more lake frozen days, which indicated a cold condition and unfavorable to fish growth. During the frozen period (from the middle of October to early April) of Lake Selincuo, the metabolism rate of Selincuo naked carp is extremely low (Chen et al. 2002b). As our data showed, the greatest number of days of annual snow accumulation during the past decade occurred in 1997 and was verified by the smallest annual growth index of Selincuo naked carp during that time. The effects of snow and sea ice also confirmed each other and have a similar mechanism (Matta et al. 2010). These results indicate that growing season is the key period for growth of *G. selincuoensis* during a typical year.

### Relationships between global climate factors and *G. selincuoensis* growth

As predicted, Selincuo naked carp growth was rarely influenced by global climate factors relevant to the Asian monsoon or the midlatitude westerlies, such as ENSO, AO, NAO, and PNA. Difference with other studies in ocean areas or near-ocean areas was due to the location of Selincuo (Black et al. 2005, 2008; Rypel 2009). This area should be affected by the Asian monsoon and the midlatitude westerlies as in other areas of Asia (Chen et al. 1991).The ENSO indirectly affects the Tibetan Plateau through the Asian monsoon (Tao and Zhang 1998; Qu et al. 2004). Due to the high elevation of the Tibetan Plateau and that Selincuo is located in the hinterland of the Tibetan Plateau, combined with the blockade by the high mountain chains of the Himalayas and Gangdise, the Asian monsoon hardly affects this area (Xu and Gao 1962; Tang and Reiter 1984; Tang 1998); thus, the influence of ENSO is negligible. AO and NAO reflect the strength of midlatitude west winds (Wallace 2000; Zhang et al. 2007), and PNA and NAO have a strong coupling effect (Timmermann et al. 1998); therefore, these factors indirectly affect Asian climate through the midlatitude westerlies. The eastward movement of the midlatitude westerlies is also resisted by the Tibetan Plateau and splits into two branches along the northern edge and the southern edge of the Tibetan Plateau (Duan and Tao 2009). As a result, the effects of these factors are also inconspicuous. The significantly correlated factors among these were April PNA and August NAO (Fig.3B), and this may contribute to weaken the monsoon and strengthen the westerlies in this area (Yao et al. 2012).

However, a notably finding in this study should not be ignored is that PDO negatively affected the growth of *G. selincuoensis* and displayed a higher interpretability than PC1 of local climate variables (Fig.4). The explanation could be PDO affects temperature and precipitation in this area (Zhu and Yang 2003). Contrary to the Pacific area (Black et al. 2008), high PDO values indicate cold conditions in Selincuo. An increasing PDO implies shortening of the growing season and decreasing temperature. Correlations among climatic factors reveal that PDO was significantly and positively correlated with ASAD and significantly and negatively correlated with temperature. Thus, the PDO index reflects a combined effect of ASAD and temperature, and showed a stronger influence on Selincuo naked carp than either of them.

Our results not only indicate that *G. selincuoensis* otoliths are suitable for developing a chronology and establishing growth–climate relationships but also the dendrochronological approach could be applied to other Schizothoracinae species and aquatic ecosystems in the Tibetan Plateau. Relationships between the *G. selincuoensis* chronology and climate factors indicate that the current climate change is beneficial to fish growth and productivity of aquatic ecosystems in the Tibetan Plateau. However, the difference in response to climate change among a wide range of species or regions on the Tibetan Plateau is still unknown and worthy of exploration. Therefore, we will conduct a dendrochronological network in the Tibetan Plateau to explore differences in specific or regional responses to climate change, even connect diverse ecosystems (aquatic and terrestrial).
